# Xerostomia, thirst, sodium gradient and inter-dialytic weight gain in hemodialysis diabetic vs. non-diabetic patients

**DOI:** 10.4317/medoral.22294

**Published:** 2018-06-21

**Authors:** Agnieszka Bruzda-Zwiech, Joanna Szczepańska, Rafał Zwiech

**Affiliations:** 1 Department of Pediatric Dentistry, Medical University of Lodz, Poland, 92-213 Lodz, Pomorska 251; 2 Dialysis Department, Norbert Barlicki Memorial Teaching Hospital No. 1 90-153 Lodz, Poland, Kopcinskiego 22

## Abstract

**Background:**

In hemodialysis (HD) patients, xerostomia and hyposalivation may intensify sensations of thirst, and contribute to the intake of fluids and excessive inter-dialytic weight gain (IWG). Since IWG is regarded to be higher in diabetic patients than in non-diabetics HD enhancing their mortality, it is crucial to define plausible underlying causes. Therefore, the study investigates factors contributing to the increased IWG in diabetic HD patients.

**Material and Methods:**

The study included 97 HD patients (38 diabetics) receiving hemodialysis. All participants completed surveys comprising the Dialysis Thirst Inventory (DTI) and Xerostomia Inventory. Unstimulated whole saliva flow rate (USWFR) was measured, with USWFR below 0.1 mL/min being regarded as hyposalivation. Additionally, pre- and post-dialysis serum sodium concentration, sodium gradient and IWG were assessed. In diabetic HD patients, hemoglobin A1c (HbA1c) level was measured.

**Results:**

Significantly higher scores were found in diabetic than non-diabetic HD patients with regard to DTI (21.2±7.7 vs. 17.1±6.2: Z=2.44, *p*=0.03) and xerostomia (40.5±6.1 vs. 29.9±14.4: Z=4.15, *p*=0.003). Hyposalivation was observed more often in diabetic HD patients (Z=2.23, *p*=0.04). IGW was significantly higher in participants with diabetes (Z=2.44, *p*=0.03), as was the pre-dialysis sodium serum (Z=3.4, *p*=0.008). High levels of HbA1c were associated with lower levels of serum sodium (r=-0.67 *p*<0.05). HbA1c positively correlated with pre-dialysis sodium gradient (r=0.66 *p*<0.05). However, multiple regression analysis found that the only predictors of increased IWG (>4.8 IWG%) in diabetic patients remained saliva flow rate and pre-dialysis sodium gradient.

**Conclusions:**

Concomitant diabetes in hemodialysis patients appears to intensify subjective xerostomia and thirst sensation. It also leads to excessive IWG by the increase of pre-dialysis serum sodium gradient.

** Key words:**Diabetes mellitus, hemodialysis, hyposalivation, inter-dialytic weight gain, sodium gradient.

## Introduction

According to the 2013 ERA-EDTA Registry annual report, diabetes is an important contributor to replacement renal therapy (RRT) in Europe and the Mediterranean. Patients with diabetes mellitus as the cause of ESRD comprised 24% of the incident RRT patients and 17% of the prevalent RRT patients (1).

Although great improvements have been noticed, patients with diabetes receiving RRT continue to have significantly worse prognosis than non-diabetic patients, as well as a higher mortality rate, which is of cardiovascular origin in the majority of cases (1,2,3).

Both diabetes mellitus and renal diseases are conditions that directly affect the salivary glands and may cause decreased salivary production (4,5). The prevalence of xerostomia, a subjective feeling of dry mouth, in hemodialysis patients ranges between 32.9 and 76.4% (6-8). It may be associated with objectively measured hyposalivation or with changes in the quality of saliva, with the amount of saliva itself remaining unchanged (9). In hemodialysis (HD) patients, xerostomia and hyposalivation lead to numerous oral sequelae (10), but may also intensify sensations of thirst, and contribute to the intake of fluids and excessive inter-dialytic weight gain (IWG); this increased intake can lead to chronic fluid overload, resulting in cardiovascular complications (6,11,12). Our previous studies found higher inter-dialysis weight gain in hemodialysis patients with hyposalivation than those with normosalivation, and a positive correlation between both xerostomia and thirst with IWG in HD patients; however, multivariable analysis found that pre-dialysis sodium gradient and lowered saliva flow rate remained significant predictors of excessive IWG. Despite this, the study did not address the hypothesis that xerostomia, hyposalivation or pre-dialysis sodium gradient contributes to greater IWG in diabetic HD patients (8).

Analysis of the United States Renal Data System (USRDS) Dialysis Morbidity and Mortality Waves 3 and 4 Study showed that large inter-dialytic weight gains of more than 4.8% are associated with shorter survival when comorbidity is taken into account (13). Owing to the fact that some studies have demonstrated that IWG is higher in diabetic HD than in HD patients without concomitant diabetes (14-16) and that increased IWG is associated with a higher mortality in diabetic patients on maintenance hemodialysis (16), it is important to define the underlying causes of increased IWG in diabetic HD patients to establish new therapeutic options. Although Sung et al. report that xerostomia and lowered serum sodium may be contributing factors to greater IGW in both diabetic and non-diabetic HD patients (14), no other previous studies have investigated whether thirst sensation or pre- and post-dialysis serum sodium gradients influence IWG in diabetic HD patients. The aim of the study was therefore to determine whether concomitant diabetes considerably influences unstimulated salivary flow rate, xerostomia, thirst sensation, serum sodium gradient and IWG in hemodialysis patients, and to investigate which of the mentioned parameters contribute to an increased IWG in diabetic HD patients.

## Material and Methods

-Study subjects

Study participants were recruited from a pool of 148 patients who were undergoing maintenance hemodialysis at the Dialysis Department of the Norbert Barlicki Memorial Teaching Hospital No. 1. The eligibility criteria for a patient to be included onto the study were as follows: age between 18 and 80 years, a fixed hemodialysis schedule of three times a week and a stable clinical condition. The exclusion criteria comprised uncontrolled hypertension or recurrent symptomatic hypotension episodes, chronic heart failure (NYHA stage 4), severe acute infections requiring hospitalization and the administration of centrally-acting sympathicolytics. Finally, the study included 97 patients (55 male and 43 female) receiving maintenance hemodialysis (mean age 59.1 ± 13.6 years), including 38 patients with diabetes. The study was conducted in compliance with the principles of the Helsinki Declaration. The study protocol was approved by the Medical University of Lodz Bioethics Committee, Resolution Number RNN 147/09/KE. Informed consent was obtained from all patients prior to their inclusion in the study. The mean time from starting hemodialysis was at least six months. The mean session time was 253 minutes. The causes of end-stage renal disease included chronic glomerulonephritis in 28 patients, diabetic nephropathy in 26, adult polycystic kidney disease in eight, hypertension in 22, tubulointerstitial nephritis in six and unknown in seven patients. All patients were advised to maintain their usual dietary habits.

Participants were divided into two subgroups: non-diabetic HD patients and diabetic HD patients with a history of diabetes longer than six months (the mean diabetic history was 38.8 ± 21.4 months). Only insulin-dependent patients participated in the study. The non-diabetic HD and the diabetic HD groups were age and sex matched, and significant parameters including number of participants with preserved residual urination, and mean hemodialysis session time were comparable. The characteristics of the non-diabetic and diabetic HD patients are summarized in  Table 1.

**Table 1:**
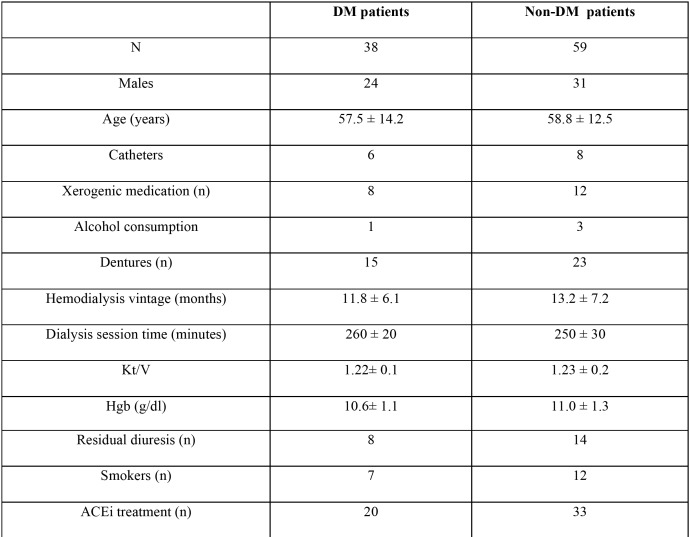
Characteristics of the study population.

-Assessment of thirst intensity, xerostomia, UWSFR, IGW and sodium gradients 

All participants completed a survey evaluating thirst intensity comprising the Dialysis Thirst Inventory (DTI) and Xerostomia Inventory (XI). The dialysis thirst inventory is a questionnaire consisting of seven items, each with a five-point Likert scale ranging from never (1) to always (5). The results ranged from a minimum of seven points (no thirst) to a maximum 35 points (enormous thirst). The validated xerostomia inventory comprises 11 items, each with a 5-point Likert scale ranging from never (1) to always (5), with the results from 11 points (no dry mouth) to a maximum 55 points (extremely dry mouth).

Unstimulated whole saliva flow rate was measured, with USWFR below 0.1 ml/min being regarded as evidence of hyposalivation (17). The unstimulated whole saliva was collected for five minutes by the spitting method before a mid-week HD session. The subject refrained from eating, tooth brushing, mouth rinsing or smoking for at least one hour before spitting. They were seated in an upright position and asked to relax during spitting. The details of the saliva collection method are described elsewhere (8). The saliva flow rate was then calculated in milliliters per minute. The survey and saliva samples were obtained under the supervision of an experienced dentist (the author) who was blinded to patients’ medical records.

The results of biochemical tests, i.e. pre- and post-dialysis serum sodium concentration and sodium gradient (the difference between serum sodium and dialysis fluid sodium concentration), are presented as absolute numbers. Hemoglobin A1c (HbA1c) was measured to assess glycemic control in HD diabetic patients. All measurements were carried out routinely, as a part of every month HD patients’ assessment, in certified central hospital laboratory automatic analyzers. At the same time, inter-dialytic weight gain (IWG), defined as the difference between current body mass and dry weight, was measured. All assessments, i.e. blood specimens, saliva collection and the survey, were conducted according to single time-point assessment during the mid-week HD session. The antihypertensive treatment allowed blood pressure below 140/90 mmHg before, and 130/80 mmHg after hemodialysis to be achieved in most of the participants. In both subgroups, antihypertensive treatment was not changed and doses were stable. The kidney replacement therapy was conducted on Fresenius 4008 dialysis machines exclusively. Standard bicarbonate dialysate fluid containing 140 mmol/l of sodium, 1.25 mmol/l of calcium and 0.75 mmol/l of magnesium was used. The potassium concentration varied depending on the degree of patient kalemia before the session. Dialysis adequacy was assessed, with a single pooled kT/V of average value 1.2 to 1.4 being regarded as adequate. Dry weight was established based on clinical examination, blood pressure measurements and whole body composition spectroscopy (18). In all participants, the mineral bone disorder associated with renal anemia and kidney diseases was successfully treated according to KDOQI and KDIGO recommendations (19,20). The above-mentioned clinical procedures were performed by the staff of the Dialysis Department and supervised by the Head of Department (co-author).

-Statistical analysis

The abnormality of the distribution was checked by the Kolmogorov-Smirnov test. Comparisons between the study subgroups were performed using the Mann-Whitney test. The Fisher’s exact probability test was used for gender comparison. Correlations were assessed by Spearman’s rank correlation coefficient. Possible predictors of excessive weight gain were analyzed by multiple regression coefficients. Differences were considered significant if the p-value was less than 0.05. The results were expressed as mean ± standard deviation and median (interquartile range). Statistical analysis was performed using Statistica for Windows software (version 10.0). Post hoc interim assessment for sample size re-estimation to asses the appropriate statistical Power test using G*Power (a free tool for test power assessment) was performed, which indicated statistical power of over 80 % (0.83).

## Results

No differences were observed between the two study groups with regard to basic parameters. The characteristics of the study population are presented in Table 1. Significantly higher scores were found in diabetic HD patients then non-diabetic patients with regard to DTI (21.2 ± 7.7 vs. 17.1 ± 6.2: Z=2.44, *p*=0.03) and xerostomia (40.5 ± 6.1 vs. 29.9 ± 14.4: Z=4.15, *p*=0.003). Although no significant difference was observed between non-diabetic HD and diabetic HD patients with regard to mean unstimulated salivary flow, hyposalivation (UWSFR<0.1 mL/min) was observed more often in diabetic HD patients (Z=2.23, *p*=0.04).

A statistically significant difference was seen between subgroups with regard to inter-dialysis weight gain, which was higher in participants with diabetes (Z=2.44, *p*=0.03). Neither pre- nor post-dialysis sodium serum concentration differed between study groups. Both HD patients with and without diabetes demonstrated similar post-dialysis sodium serum gradients. However, the pre-dialysis sodium serum gradient was significantly higher in the group with diabetes (Z=3.4, *p*=0.008).  Table 2 presents the comparison of described parameters in HD patients with and without diabetes. In all DM-patients, hemoglobin A1c (HbA1c) level was measured to assess glycemic control. Its mean value was stable at 6.5 ± 0.4 %. Higher levels of HbA1c were associated with lower levels of serum sodium (r= -0.67 *p*<0.05). HbA1c level positively correlated with pre-dialysis sodium serum gradient (r=0.66 *p*<0.05).  Table 3 shows the results of multiple regression of predictors (coefficients) of excessive weight gain (>4.8 IWG%) in DM hemodialysis patients. The only predictors of increased IWG in diabetic patients were saliva flow rate and pre-dialysis sodium gradient.

**Table 2:**
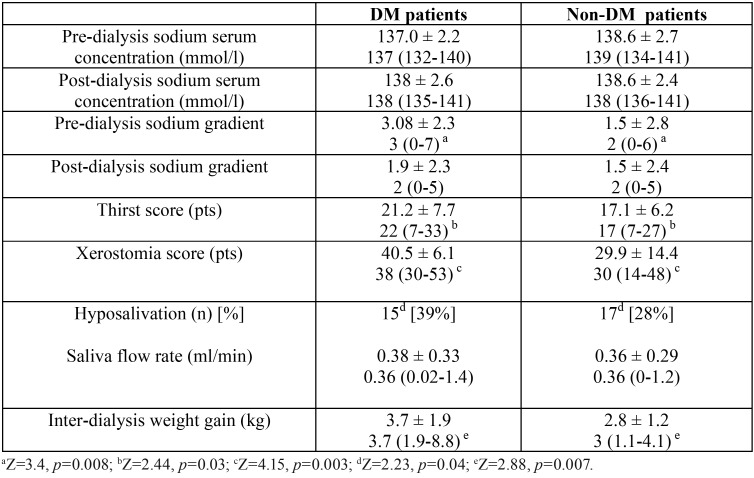
The comparison of parameters in HD patients with (DM patients) and without diabetes mellitus (Non-DM patients).

**Table 3:**
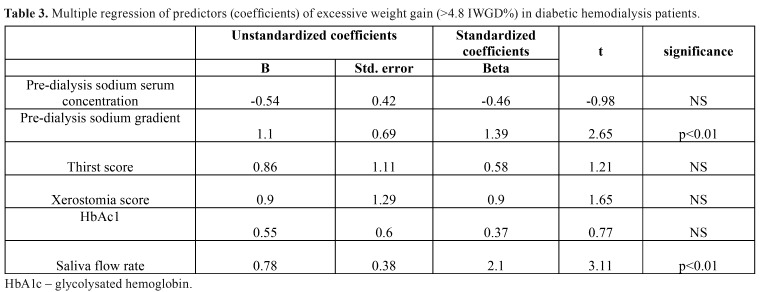
Multiple regression of predictors (coefficients) of excessive weight gain (>4.8 IWGD%) in diabetic hemodialysis patients.

## Discussion

The results of our study confirm that xerostomia is more severe in hemodialysis patients with insulin-dependent diabetes when assessed using the Xerostomia Inventory. This was also noted by Chuang *et al.* (20,21) and Sung *et al.* (14), who evaluated the severity of dry mouth sensation in HD patients with or without diabetes using a visual analog scale (VAS). In addition, Swapna *et al.* report that xerostomia is more common in HD patients suffering from diabetes - 62% of non-sufferers vs. 78.7% of sufferers (22). In contrast to Sung *et al.* (14), our findings do not indicate any significant differences in UWSFR between diabetic and non-diabetic HD patients. This could be explained by the fact that in ERDS patients on maintenance hemodialysis, uremic involvement of salivary glands and dehydration from restricted fluid intake can lead to decreased salivary flow. However, hyposalivation (USWFR below 0.1 ml/min) was observed more often in diabetic HD patients, probably due to the synergic effect of diabetes and uremic changes in salivary glands. Also, Vesterinen *et al.* found no differences in USWFR in cases of diabetic and non-diabetic chronic kidney disease at the predialysis stage (0.4±0.9 ml/min vs. 0.2±0.9 ml/min); however, stimulated salivary flow was significantly lowered in the diabetic group (23). In contrast, Teratani *et al.* report no statistically significant differences in stimulated salivary flow in patients undergoing HD for diabetic nephropathy or chronic glomerulonephritis, although the saliva flow rate was significantly lower in both groups of HD patients than in the control group (24). Our findings confirm that inter-dialytic weight gains are higher in diabetic HD patients than those without concomitant diabetes, which is in line with previous studies (14-16). One of predictors of increased IWG in diabetic HD patient was lowered saliva flow (UWSFR). In addition, Sung *et al.* who examined the interaction between UWSFR and IGW in study group numerically similar to ours -51 non-diabetic and 40 diabetic HD patients, note that UWSFR was negatively correlated with IWG in diabetic and non-diabetic HD patients (14).

However, in contrast to Sung *et al.* (14), who found oral dryness score to be associated with IWG in both non-diabetic and diabetic HD patients, the only predictor in diabetic HD patients in the present study was found to be pre-dialysis sodium gradient. In addition, our findings show that diabetic HD patients with higher levels of HbA1c had lower serum sodium levels and higher pre-dialysis sodium gradients, and Creame and Mc Caffery report a negative correlation between poor glycemic control and pre-dialysis sodium (25). Those findings seem to provide a new insight into the mechanisms behind excessive IGW in diabetic patients.

IWG is the product of accumulation of water in the body from the metabolism and by dietary and fluid intake. The major mechanism leading to excessive IWG in HD patients seems to be the osmoregulation pathway (cit. 14). In our study, diabetic HD patients were found to have a higher thirst score. Thirst is induced when osmoreceptors are stimulated by high plasma osmolality. However, Thompson *et al.* found hypertonic D-glucose infusion to cause similar elevations in blood glucose in diabetic patients and healthy controls, but did not change plasma vasopressin or thirst ratings; in contrast, infusion of hypertonic sodium chloride was accompanied by progressive increases in plasma vasopressin in both diabetic and non-diabetic patients, implying that hyperglycemia per se is not a direct stimulus of thirst in diabetic patients (26).

Not complying with salt restriction, followed by excessive water intake, is believed to be one of the key causes of increased IWG in hemodialysis patients. To maintain stable osmolality under physiological conditions, excessive sodium urine excretion is triggered. However, this mechanism is insufficient in HD diabetic patients, even those with residual diuresis, and fluid intake is the only mechanism that can restore normal osmolality. Since water cannot be effectively excreted in HD patients, excess fluid intake causes hypo-osmolality, which in turn increases salt appetite and sodium intake. Therefore, HD patients not complying with salt restriction might demonstrate greater IGW without hyponatremia, whereas patients complying with salt restriction by dilutional effect due to excessive water intake might have greater IWG with hyponatremia, due to the dilutional effect caused by excessive water intake (cit. 14). Hence, the presence of a lower serum sodium concentration in diabetic HD patients than non-diabetic HD patients, observed in our present study, may suggest that the greater IGW found in these groups of patients was not only due to more salt consumption, which is a natural thirst stimulus, but that other stimuli were involved. Therefore, patients with high inter-dialytic weight gain and low predialysis sodium should be assessed for other reasons for fluid intake, such as high blood glucose or social drinking (27). Hyperglycemia increases extracellular fluid osmolality and causes an osmotic shift of water and potassium from the intracellular to the extracellular fluid (2). Water translocation induced by elevated serum glucose level results in a relative decrease in serum sodium concentration, i.e. translocational hyponatremia (28). Synergic effect of hyperglycemia and non-effective sodium elimination probably provoke thirst, as fluid intake is the only mechanism by which normal osmolality can be restored. It could also explain the significantly higher thirst score in diabetic HD patients than non-diabeticpatients observed in our study. In diabetic HD patients, excessive fluid intake leads to fluid overload, resulting in lowered serum sodium and increased pre-dialysis sodium gradient. As noted in our previous study, increased pre-dialysis sodium gradients might be a contributory factor to increased IWG in HD patients. The described mechanism seems to create a ‘vicious circle’ effect, which is most clearly observed in diabetic patients. The presence of elevated IWG caused by increased fluid gains implicates a higher ultrafiltration rate during hemodialysis session. Nevertheless, rapid body fluid shifts, mainly in the extracellular intravascular compartment, results in intra-dialysis hypotension. In diabetes patients, deregulation of the autonomic nervous system caused by ERDS (uremia) is further aggravated by diabetic neuropathy, resulting in intra-dialysis hypotension (29). This results in a failure to maintain an adequate ultrafiltration rate and to achieve an ideal dry body mass, leading to persistent fluid overload and a lowering of serum sodium in diabetic HD patients. Good glycemic control is therefore of great importance in diabetic HD patients, as studies of Hwang *et al.* (29), Mandai *et al.* (30), Perez-Garcia (31) demonstrate that lower serum sodium is an independent predictor of a higher risk infection-related hospitalization, and is associated with a high prevalence of comorbidities and long-term mortality risk in patients on maintenance hemodialysis.

In conclusion, concomitant diabetes in hemodialysis patients appears to intensify subjective xerostomia and thirst sensation. It also leads to excessive inter-dialytic weight gain by the increase of pre-dialysis serum sodium gradients.
